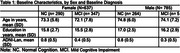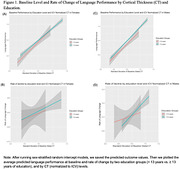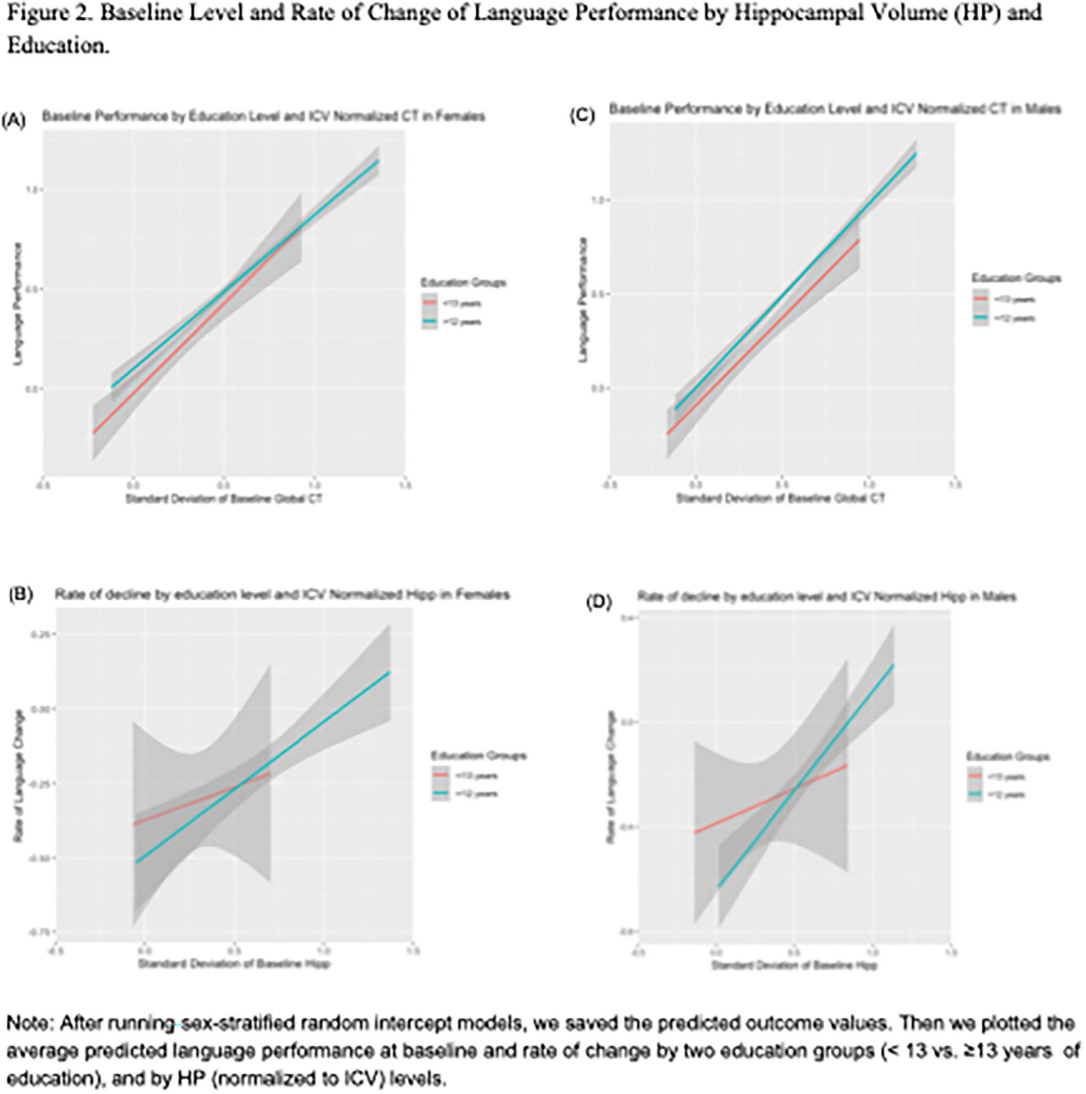# The Role of Education on Brain Pathology and Cognitive Decline in the Language Domain Across Women and Men in the Alzheimer’s Disease Neuroimaging Initiative Sample

**DOI:** 10.1002/alz.095686

**Published:** 2025-01-09

**Authors:** Min Hee Kim, Gelan Ying, Brittany T Morin, Alice Gavarrete Olvera, Laura E. Gibbons, Phoebe Scollard, Michael L. Lee, Brandon S Klinedinst, Connie Nakano, Jesse Mez, Paul K. Crane, ADNI, Seo‐Eun Choi

**Affiliations:** ^1^ University of California San Francisco, San Francisco, CA USA; ^2^ University of Florida, Gainesville, FL USA; ^3^ University of California San Francisco (UCSF), San Francisco, CA USA; ^4^ Columbia University Medical Center, CUNY Graduate Center & Queens College, New York, NY USA; ^5^ Department of Medicine, University of Washington, Seattle, WA USA; ^6^ University of Washington, School of Medicine, Seattle, WA USA; ^7^ Department of Neurology, Boston University Chobanian & Avedisian School of Medicine, Boston, MA USA; ^8^ Alzheimer’s Disease Neuroimaging Initiative, http://adni.loni.usc.edu/, CA USA

## Abstract

**Background:**

Education is a strong predictor influencing the dementia progression. With diminished brain integrity, cognitive reserve (CR) is thought to help preserve cognitive function and delay the symptom manifestation. Yet, scholars have not reached consensus on the extent to which education modifies brain integrity‐cognitive decline associations and how it differs by sex/gender.

**Method:**

To test the hypothesis about CR on cognitive decline, we used the Alzheimer’s Disease Neuroimaging Initiative (ADNI) sample and included 785 male and 637 female participants, who were cognitively normal or had mild cognitive impairment at baseline (Table 1). We used three global brain integrity measures (cortical thickness (CT), hippocampal volume (HP), and Reversed‐White Matter Hyperintensities (R‐WMH) as well as the language composite score using the item response theory. We applied sex‐stratified random intercept models including the three‐way interaction term (*education* x *CT* x *time)* against the 5‐year language performance. A robust set of contextual confounders was included; intracranial volume (ICV) difference was accounted for in two ways: covariate adjustment vs. normalization.

**Results:**

The average age was 72.6 (7.0 SD) and 74.3 years (6.8 SD) for female and males, respectively. Males had high average years of education than females. The linear regression models showed that high levels of brain integrity (HP, CT) were associated with high baseline performance and slower decline; education was only associated with higher baseline performance. The estimation of education modifying the association between brain integrity and language was sensitive to ICV correction methods and differed by brain integrity type and sex. For example, education attenuated the relationship between low normalized‐CT and R‐WMH measures and faster decline (i.e., the lower CT or WMH and decline association was weaker with higher education) only in female sample (Figure 1B); such association was not hold when ICV was included as covariate. In both males and females, lower HP and decline association was stronger with higher education, regardless ICV correction methods (Figure 2).

**Conclusion:**

Incongruence between theory and empirical evidence on education and cognitive reserve may stem from which brain integrity measures are used, and the different approaches to account for ICV.